# The Process and Regulatory Components of Inflammation in Brain Oncogenesis

**DOI:** 10.3390/biom7020034

**Published:** 2017-03-27

**Authors:** A.G.M. Mostofa, Surendra R. Punganuru, Hanumantha Rao Madala, Mohammad Al-Obaide, Kalkunte S. Srivenugopal

**Affiliations:** Department of Biomedical Sciences and Cancer Biology Center, School of Pharmacy, Texas Tech University Health Sciences Center, 1406 S. Coulter St., Amarillo, TX 79106, USA; agm.mostofa@ttuhsc.edu (A.G.M.M.); Surendra.r.punganuru@ttuhsc.edu (S.R.P.); hanumantharao.madala@ttuhsc.edu (H.R.M.); Mohammad.al-obaide@ttuhsc.edu (M.A.-O.)

**Keywords:** inflammation, gliomas, interleukins, STAT3, epigenetic deregulation, MGMT DNA repair

## Abstract

Central nervous system tumors comprising the primary cancers and brain metastases remain the most lethal neoplasms and challenging to treat. Substantial evidence points to a paramount role for inflammation in the pathology leading to gliomagenesis, malignant progression and tumor aggressiveness in the central nervous system (CNS) microenvironment. This review summarizes the salient contributions of oxidative stress, interleukins, tumor necrosis factor-α(TNF-α), cyclooxygenases, and transcription factors such as signal transducer and activator of transcription 3 (STAT3) and nuclear factor kappa-light-chain-enhancer of activated B-cells (NF-κB) and the associated cross-talks to the inflammatory signaling in CNS cancers. The roles of reactive astrocytes, tumor associated microglia and macrophages, metabolic alterations, microsatellite instability, *O*^6^-methylguanine DNA methyltransferase (MGMT) DNA repair and epigenetic alterations mediated by the isocitrate dehydrogenase 1 (IDH1) mutations have been discussed. The inflammatory pathways with relevance to the brain cancer treatments have been highlighted.

## 1. Introduction

Primary brain tumors are most common malignancies (85–90%) of the central nervous system (CNS) [1]. Many of these cancers pose unique challenges to treat and cause significant mortality. There are many different types and subtypes: gliomas (e.g., astrocytomas, oligodendrogliomas, ependymomas) and non-glial tumors (e.g., meningiomas, medulloblastomas). On the basis of histology, all brain tumors are classified into four grades: Grade I to IV. Grade I show low proliferative potential and can be cured surgically, Grade II exhibits some extent of infiltration and recurrence after surgery, and Grade III tumors demonstrate more atypia and mitotic activity, Grade IV tumors are the most malignant with vascular proliferation and necrosis [2]. The glioblastoma multiforme (GBM), a grade IV tumor accounts for 80% of all primary brain cancers. The CNS is also an important site for lodging metastatic cancers from other organs. Around 40% of all non-brain cancers, particularly the non-small cell lung cancer (NSCLC), breast cancer, and melanoma eventually metastasize to the brain [3]. The incidence of CNS malignancies is increasing in the United States and around the world. Current treatment options for brain tumors include surgery, radiation and chemotherapy. Due to limited chemotherapeutic alternatives (mainly the methylating and chloro-ethylating alkylating agents), the overall survival rate and 5-years relative survival rate of high-grade glioma are approximately 17% and 6% respectively [1]. These statistics are much worse for patients with metastasis to the brain (2-years survival rate is 8%) [4].

Like any other malignancy, the development of brain cancers is a multistep process which begins with genetic alterations in precancerous cells. Numerous genetic mutations identified in brain cancers include the tumor protein p53 (TP53), phosphatase and tensin homolog (PTEN), neurofibromatosis type 1 (NF1), epidermal growth factor receptor (EGFR), retinoblastoma (RB), and phosphoinositide-3-kinase regulatory subunit 1 (PIK3R1) [5]. Most of these genes encode proteins with tumor suppressing functions. Homozygous p16 gene deletion is another important genetic feature in many brain cancers, especially in higher-grade tumors [6]. Cyclin-dependent kinase inhibitor 2A (p16) is a cell cycle inhibitor protein that prevents abnormal cell growth and proliferation by binding to cyclin-D and cyclin-dependent kinases 4 or 6 (CDK4 or CDK6) complexes. Understanding the impact of these diverse gene mutations and/or deletions in various signaling pathways is crucial to reflect on about the biological properties and pathogenic characteristics of different brain cancers. To date, alterations of four major pathways have been found in brain cancers, where at least one member of the signaling or metabolic circuit is mutated: RTK (receptor tyrosine kinases)/RAS (rat sarcoma)/PI3K (phosphotidylinositol-3-kinase) pathway, p53 pathway, RB pathway and the isocitrate dehydrogenase 1 or 2 (IDH1/2) pathway [7,8]. All these alterations confer carcinogenic characteristics in cells by several key changes that include promotion of growth signals, non-responsiveness to antigrowth signals, evasion from apoptosis and acquisition of metastatic potential. Identification of somatic mutations in the key Krebs cycle enzymes, namely, the IDH1/2 in low-grade gliomas (astrocytomas, oligodendrogliomas, oligoastrocytomas) and secondary glioblastomas, but not in primary glioblastomas has been a recent focal point in glioma biology [9]. Heterozygous IDH mutant enzymes generate the oncometabolite d-2-hydoxyglutarate (d-2HG) instead of the normal α-ketoglutarate (α-KG) in the citric acid cycle [10]. d-2HG appears to promote gliomagenesis through two distinct mechanisms; one by activating nuclear translocation of hypoxia-inducible factor (HIF)-1 which causes transcriptional activation of genes leading to abnormal cell proliferation and angiogenesis and second, by restructuring the cellular epigenetic state through a global increase of cellular DNA and histone hypermethylation [9,11]. The mechanistic aspects of d-2HG in glioma epigenetic alterations are summarized later in this review.

Epigenetic deregulation indeed plays a critical role in neoplastic transformation and malignant progression of brain cancers. These can be of various types, including DNA methylation, histone posttranslational modifications, long-noncoding RNAs (lncRNAs) and micro RNAs (miRNAs) [12]. DNA methylation of CpG islands (hypermethylation) in promoter regions causes silencing of tumor suppressor genes. Deregulated expression of histone modifying enzymes alters chromatin state and transcription. miRNAs can also play a role in tumorigenesis through targeting gene expression at messenger RNA (mRNA) level. Many of these epimutations have been identified in brain cancers that may trigger suppression of tumor inhibitory genes or over-activation of tumor promoting factors [13].

The connection between inflammation and cancer is a well-established concept, and now it is considered to be one of the hallmarks of cancer [14]. Inflammatory components are ubiquitously found in the microenvironment of various neoplastic tissues. Cancer-related inflammation manifests several key features: infiltration of white blood cells (e.g., tumor-associated macrophages), the presence of inflammatory mediators (e.g., cytokines, interleukins and chemokines), development of vascular growth and tissue remodeling. Although a well-regulated inflammatory response should exert an anti-tumorigenic effect, ironically chronic inflammation leads in the opposite direction. It predisposes cells for an oncogenic transformation and molecular alterations through both extrinsic and intrinsic pathways. In the intrinsic pathway, carcinogenesis associated genetic events initiate inflammation-related programs that guide for the development of an inflammatory microenvironment. In the extrinsic pathway, chronic inflammation acts as a driving force to facilitate cancer development. In this review, we discuss the inflammatory microenvironment in brain tumors, inflammation-induced genetic and epigenetic alterations and the role of the inflammatory mediators in brain cancer progression. Elucidation of such interactive effects of inflammation on brain cancers is expected to provide an opportunity to identify novel targets for better diagnosis and treatment.

## 2. Inflammatory Microenvironment in Brain Tumor

**Figure 1:**
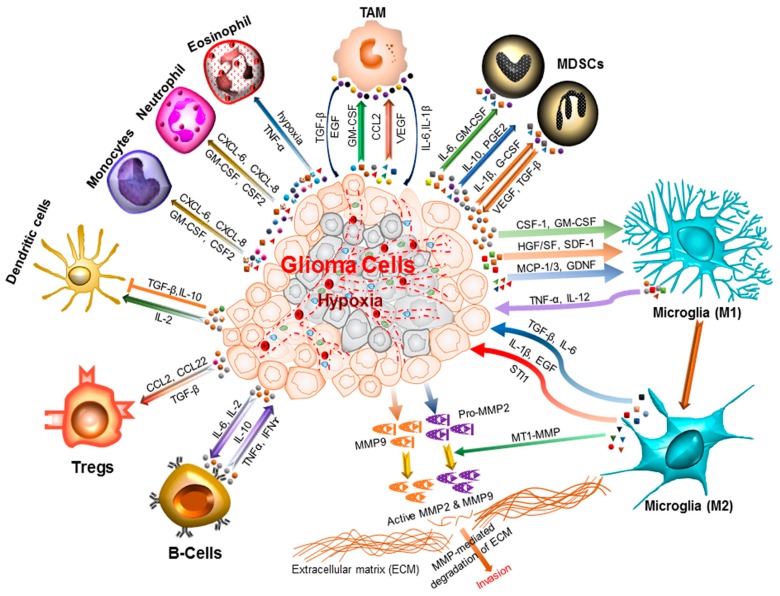
Inflammatory microenvironment prevalent in brain cancers. The glioma microenvironment is heavily infiltrated with different inflammatory cells, including microglia, macrophage, neutrophil, eosinophil, monocytes, dendritic cells, T-cells, B-cells, and myeloid derived suppressor cells (MDSCs) [18]. Upon activation, these cells release an array of mediators that promote cancer cell proliferation, survival, migration and invasion. These include the pro-inflammatory and cytotoxic cytokines, growth factors, bioactive lipids, hydrolytic enzymes, matrix metalloproteinases, reactive oxygen intermediates, and nitric oxide [19,20]. The cell types involved and the cytokines produced are shown. CCL: C–C Motif Chemokine Ligand 2/monocyte chemoattractant protein 1 (MCP1); TNF-α: Tumor necrosis factor-alpha; CXCL: Chemokine (C–X–C motif) ligand; INF-ɣ: Interferon gamma; TAM: Tumor-associated macrophages; Tregs: Regulatory T-cells; IL-1β: Interleukin 1 beta; IL-2/6/10/12: Interleukin 2/6/10/12; CSF-1: Colony stimulating factor 1; GM-CSF: Granulocyte-macrophage colony-stimulating factor; HGF/SF: Hepatocyte growth factor or scatter factor; SDF-1: Stromal cell-derived factor 1; MCP-1/3 : Monocyte chemoattractant protein 1 or 3; GDNF: Glial cell-derived neurotrophic factor; TGF-β: Transforming growth factor beta; EGF: Epidermal growth factor; STI1: Stress inducible protein 1; MT1-MMP: Membrane type 1-matrix metalloproteinase; MMP2/9: Matrix metalloproteinase 2 or 9.

The brain tumor microenvironment is composed of different types of cells, including infiltrative inflammatory cells, cells with stem-like properties, and cells with neural, glial and myeloid markers (Figure 1). Hypoxia and abnormal vascular proliferation occur due to excessive tumor growth that causes infiltration of various immune cells such as macrophages, eosinophils, neutrophils and T-lymphocytes [15]. Two types of astrocytes contributing to inflammation (A1 and A2) in glioma niche [16,17] have been identified. While the A2 subtype supports neuronal survival during oxygen deprivation and maintains neuronal connectivity, the A1 reactive astrocytes appear neurotoxic in nature and mainly formed in response to injury and pro-inflammatory factors. A crosstalk between reactive A1 astrocytes and resident glioma cells that promote localized inflammation has been suggested [17].

There is a complex crosstalk between these different cell types that generate numerous cytokines (Figure 1). These cytokines function in both paracrine and autocrine manner resulting in activation or suppression of multiple signaling pathways that primarily evolved to facilitate tissue repair. However, the chronic inflammatory condition of tumor microenvironment manipulates those signals in such ways that promote tumor proliferation and metastasis.

The primary function of the inflammatory mediators is to clean up unwanted cells present and to promote fibrous tissue growth to replenish the injured area. However, in the state of chronic injury, inflammatory cells react in a different way by secreting higher levels of immune inhibitory cytokines and other immunosuppressants. Similarly, the tumor-infiltrating immune cells like cytotoxic T-cells and natural killer cells start producing inflammatory mediators instead of exhibiting tumoricidal activity [21]. Therefore, the inflammatory microenvironment induces dysregulated immune response and facilitates tumor progression in precancerous lesions.

The microglial cells and tumor associated macrophages (TAM) are two ubiquitous components of brain tumor microenvironment that accumulate in and around glioma tissue and cooperate in cancer cell proliferation and invasion. Microglia and TAM recruitment is mediated through various cytokines and chemoattractant factors such as the monocyte chemoattractant protein-1 and 3 (MCP-1, MCP-3), hepatocyte growth factor and scatter factor (HGF/SF), granulocyte macrophage colony-stimulating factor (GM-CSF), macrophage colony-stimulating factor-1 (CSF-1), glial cell-derived neurotrophic factor (GDNF), as shown in Figure 1. Glioma-associated microglia and macrophages release several cytokines and growth factors (interleukin (IL) IL-6, IL-1β, transforming growth factor-β (TGF-β)), epidermal growth factor (EGF), and stress-inducible protein 1 (STI1) that facilitate the tumor proliferation and migration. The TGF-β isoform TGF-β2 induces degradative enzymes matrix metalloproteases (MMP2 and MMP9) that upon activation by membrane type 1-matrix metalloproteinase (MT1-MMP) and urokinase plasminogen activator (uPA) degrade the extracellular matrix to promote glioma invasion [22]. Glioma cells release MMPs as inactive zymogens and increased expression of these enzymes in this cancer type has been well documented [23,24]. MDSCs present in glioma microenvironment mediate immunosuppression by interfering with the induction of cytotoxic T-cell reactivity. MDSCs may also promote angiogenesis and metastasis through secretion of various factors such as vascular endothelial growth factor (VEGF) and GM-CSF [25,26]. Several other cell types (leukocytes, humoral and adaptive immune cells) have also identified in brain tumor niche that participate in the cross-talk with glioma cells. Immune inhibitory cytokines maintain an immunosuppressive environment at the tumor site. Further, the hypoxia and abnormal vascular proliferation resulting from continued tumor growth can trigger the induction of several inflammatory mediators and promote microglia activation as shown in Figure 1.

Metastasis of the breast cancers, non-small cell lung cancers and melanoma to the brain is also facilitated by the inflammatory mediators like T-cells and tumor associated macrophages [3]. To metastasize, brain metastasis initiating cells first need to detach from the original sites and invade into systemic circulation. Presence of macrophages in the tumor microenvironment enhances cancer cell intravasation through formation of actin-rich degradation protrusion followed by degradation of matrix barriers [27]. Upon reaching the circulation, the BM initiating cells have to evade from immune attack. Inflammation-induced deregulated immune response assists cancer cells to safely a passage through circulation [28]. The resident cytokines and chemokines produced by preexisting inflammatory brain, contribute to the brain-specific metastatic behavior of cancer cells by promoting adhesion to the brain vasculature and subsequent migration through the microvascular endothelial cells [29]. During extravasation, inflammatory macrophage and myeloid derived suppressor cells assist by invading cancer cells and promoting angiogenesis and remodeling of the extracellular matrix.

## 3. Tumor Associated Microglia and Macrophages

Microglia or glioma associated macrophages (GAM) are the most common myeloid-derived cells present in brain tumor microenvironment that either arises from resident CNS macrophages or from circulating monocytes through differentiation [30]. GAMs comprise approximately 30% of tumor inflammatory cells and Glioma and GAMs cooperate in multiple paracrine networks that facilitate their coexistence [20]. For instance, Glioma cells stimulate microglia invasion through the production of various factors (chemokines and cytokines) that intrigue the macrophages in the tumor site (Figure 1). Similarly, GAMs activate different tumorigenic pathways and thereby assist in cancer progression.

Several chemokines were reported to be involved in GAM chemo-attraction, including monocyte chemotactic protein-1(MCP-1)/C–C motif chemokine ligand 2(CCL2), C–C motif chemokine ligand 5 (CCL5), monocyte chemoattractant protein (MCP-3)/C–C motif chemokine ligand 7 (CCL7) [31]. These chemokines are not normally expressed in brain cells but appear during tumorigenic condition. Their expression in gliomas was shown to have significant prognostic value and correlated to the tumor stage. Stromal-derived factor-1 (SDF-1/CXCL12) is another chemokine produced by hypoxic and invasive brain tumors that promote GAM recruitment in glioma microenvironment [32,33]. Its knockdown significantly reduces macrophage infiltration in hypoxic regions observed in a murine brain tumor model. Other macrophage trafficking factors are CSF-1, tumor necrosis factor of the mouse embryo (TROY) and chemokine C–X–C motif ligand 1 (CX3CL1) [34,35,36]. Many studies have shown increased expression of these factors in various brain cancers and correlate with advanced disease and progression.

Cytokines have a pivotal role in the regulation of macrophage phenotype which is distinguished into two different spectrum: M1 and M2. Some cytokines convert GAMs to classically activated form which is M1 phenotype while others cause M2 transformation (Figure 1). These two phenotypes affect tumorigenesis distinctively. M1 macrophages show pro-inflammatory activity and release cytotoxic factors like IL-12 and TNF-α. In contrast, alternatively activated GAMs (M2 macrophages) exert immunosuppressive and tumor promoting characteristics through secretion of immunosuppressive cytokines such as IL-10 and TGF-β [37]. These macrophages can also release growth factors like VEGF and proteases like MMP2 and MMP9 [38].

## 4. Cytokine Function in Inflammation and Brain Cancer

Cytokines are soluble mediators of innate and adaptive immune response that are released by various cells in response to pathogens or tissue damage. These are small molecules, mainly composed of glycoproteins and polypeptides, with a broad range of functions. Depending on upstream signaling and surrounding conditions cytokines may exert both pro-inflammatory and anti-inflammatory effects (Table 1). They are the major components of cellular inflammatory responses and work on removing any unwanted objects from the tissue to maintain homeostasis. In the normal physiological context, after tissue repair or pathogen elimination, inflammation is resolved, and the homeostatic state is recovered. However, if the system cannot adequately fix the cause of inflammation, a chronic pathogenic condition ensues. The role of chronic inflammation in cancer pathogenesis is now a widely accepted fact. Recent studies have discovered the molecular mechanism of inflammation mediated cancer progression where cytokines are acting as key players. Cytokines can directly serve as effectors of several tumorigenic processes, including communication between tumor cells and stromal cells, recruitment of immune cells in the tumor microenvironment, cellular differentiation, cell migration and invasion, and immune evasion [39]. Human malignant brain tumor specimens including glioma, neuroblastoma, and medulloblastoma express a high level of different cytokines that are involved in multiple pathways of cancer progression [40]. Some key cytokines that are known to participate in brain cancer progression (Table 1) are discussed under the sections to follow.

### 4.1. Interleukin-6 (IL-6)

IL-6 is an important cytokine that is secreted by neurons, microglia, astrocytes and peripheral monocytes [41]. It is involved in T helper 2 (Th2) cell response and overexpressed in various brain cancers along with its receptor. Some important physiological functions of IL-6 include neuroprotection, reactive astrogliosis and pathological inflammatory response (Table 1). It also plays a pivotal role in B cell maturation and neurogenesis upon tissue injury. Samaras et al. found that IL-6 secretion is significantly higher in peripheral monocytes of glioma patients than in control patients [41]. Authors have shown localized expression of IL-6 in tumor cells, macrophages, and ischemic necrosis by using immunohistochemistry (IHC) staining. IL-6 is found to be involved in the etiopathology of many neurological diseases as well such as Parkinson’s and Alzheimer’s disease. Some other brain conditions, including infection, traumatic brain injury and ischemia are also affected by IL-6 [42].

Evidently, IL6 has an important association with brain cancer development. Upon secretion from astrocytes, IL-6 facilitates tumor progression through induction of angiogenesis, cell proliferation and resistance to apoptosis [43]. One study has reported IL-6 mediated promotion of GBM cell invasiveness [44]. Another study found a compelling correlation between IL-6 mRNA level and extent of glioma malignancy [45]; this study also demonstrated a significant inhibition of brain tumor development upon inhibition of IL-6 signaling. Tenidap, a novel antirheumatic agent, showed remarkable inhibition of astrocytoma tumor formation in another study [46]. Later it was found that Tenidap acts a potent inhibitor of cytokine production, including IL-6. Due to its indispensable role in glioma development, IL-6 is a promising target for immunotherapy in GBM treatment.

### 4.2. Interleukin-8 (IL-8)

IL-8, a potent mediator of angiogenesis, is highly overexpressed in most brain cancers [47]. IL-8 induces the production of MMPs that play a crucial role in angiogenesis. It also counters the apoptotic death of endothelial cells which in turn produces more MMPs. Another important role of IL-8 is its chemotactic attraction of different leukocytes, especially neutrophil, which exemplifies its involvement in various inflammatory responses and infectious disease. Upon secretion by monocytes and macrophages, IL-8 promotes migration of neutrophils, basophils, and T lymphocytes (Table 1).

Macrophages massively infiltrate brain tumor microenvironment, and its density correlates with tumor the grade in GBM. Hence, IL-8 is mainly present in the perivascular areas of pseudopalisading cells near the necrosis. High-grade gliomas show increased expression of IL-8. As a tightly regulated cytokine, the endogenous expression of IL-8 is very low or almost undetectable in normal CNS area. In most cases, other pro-inflammatory cytokines induce its expression. Carlsson et al. examined the expression of IL-8 in patients with GBM and found IL-8 and VEGF to be potent angiogenic factors for progression of malignant tumors [48]. IL-8 is specifically associated with neovascularization while correlating with the histopathological grade of gliomas.

The aberrant expression of IL-8 in GBM is thought to be caused by the activation of NF-κB. One study found that expression of IL-8 enhanced the invasiveness of GBM cells and an IL-8 neutralizing antibody can remarkably reduce the invasive potential in comparison to the control IgG antibody administration [49]. Another study performed through Matrigel analysis showed marked reduction of cellular invasive potential upon down-regulation of IL-8 by short interfering RNA (siRNA) [50]. These studies indicate an integral role for IL-8 in GBM invasiveness and angiogenesis (Table 1).

### 4.3. Interleukin-1-β (IL-1β)

IL-1β, a member of the IL-1 family, is a pro-inflammatory cytokine involved in the pathogenesis of several inflammatory diseases [51]. IL-1β is constitutively produced in astrocytomas and other brain tumors and can contribute to tumor growth and metastasis [52]. It induces secretion of other pro-inflammatory cytokines and growth factors in astrocytoma and influence astrocytoma cell function and growth (Table 1). IL-1β can also cause NF-κB activation through IκB kinase (IKK) phosphorylation and its subsequent degradation [53]. Moreover, IL-1β can cause induction of Cyclooxygenase-2 (COX-2) and Prostaglandin E2 (PGE2) biosynthesis [54]. Treatment of astroglial cells with IL-1β lead to activation of extracellular signal-regulated kinase (ERK1/2) and mitogen-activated protein kinase (MAPK) [55]. Paugh et al. showed that IL-1β induces Sphingosine kinase-1 upregulation that results in increased tumor cell survival and invasiveness [53]. In another study, Wang et al. found high levels of IL-1β in the glioma microenvironment and its association with TGF-β in neurosphere formation through upregulation of stemness factor genes [56].

### 4.4. Macrophage Migration Inhibitory Factor (MIF)

MIF was originally discovered as a T-cell product that inhibits migration of macrophages [57]. Later it was revealed that MIF could act as a pro-inflammatory cytokine and take part in the regulation of immune and inflammatory responses [58]. It has functions in the pathophysiological process as well. The inflammatory function of MIF is mediated through enhanced expression of Toll-like receptor-4 (TLR4) and increased production of inflammatory cytokines including IL-6, IL-1, and TNF-α [59]. Recent studies have confirmed the overexpression of MIF in different cancer including brain cancer. Fukaya et al. reported that the brain tumor initiating cells (BTIC) exhibit significantly higher MIF expression than non-brain tumor initiating cells (nBTIC) [60]. Furthermore, the authors found a novel p53 regulating function of MIF where MIF directly acts as intracellular p53 inactivator and thereby affect cell proliferation and apoptosis in brain cancer cells. MIF can trigger the release of the main angiogenic factors through CD44, CD74, and MAPK signaling pathways [61]. Munaut et al. demonstrated a strong correlation between MIF and VEGF expression in human glioblastoma [62]. Recently Funamizu et al. observed that MIF could induce the epithelial to mesenchymal transition (EMT), which is believed to contribute to tumor aggressiveness and chemotherapy resistance (Table 1).

### 4.5. Tumor Necrosis Factor-α (TNF-α)

TNF-α is a pro-inflammatory cytokine secreted by monocytes, macrophages, activated natural killer (NK) cells and T-cells. It has two distinct receptors, TNF-receptor 1 (TNF-R1) expressed in all cell types and contains a death domain that activates a pathway involved in apoptosis, and TNF-receptor 2 (TNF-R2) only expressed on hematopoietic cells and lacked the death domain. In a healthy brain, TNF-α is responsible for dendritic cell (DC) maturation whereas, in tumorigenic conditions its expression correlates with GBM tumor grade [63]. 80% of anaplastic GBM tumors and 17% of astrocytoma and oligoastrocytoma express TNF-α [64]. Although TNF-α was shown to have toxic effects on tumor cells at high doses, it demonstrated a tumor-promoting role in pre-clinical studies [65]. The primary receptor that mediates the cytotoxic effect of TNF-α is TNFR1. In glioma microenvironment TNF-α secretion lead to promotion tumor formation and angiogenesis [66]. It produces neovascularization through the induction of VEGF and IL-8. Tanabe et al. have found that TNF-α can instigate phosphorylation of NF-κB and signal transducer and activator of transcription 3 (STAT3) which lead to increased release of IL-6 in tumor site [67]. Another study demonstrated TNF-α induced increase in major histocompatibility complex class I (MHC-I) expression and transcriptional activation which was concurrent with increased HIF-1α, NF-κB, and β-catenin activities [68]. Thus, TNF-α enables glioma cells to escape from immune response and grow aggressively in the inflammatory microenvironment. TNF-α also plays a significant role in tumor growth by activating macrophages through SDF-1 induction to attack T-cells and other immunogenic factors (Table 1).

### 4.6. Transforming Growth Factor-β (TGF-β)

TGF-β is one of the best-characterized cytokines in brain cancers. It has three receptors that activate the transcription factors (e.g., mothers against decapentaplegic homolog or SMADs), which in turn activate various downstream genes that are involved in angiogenesis and migration/invasion such as VEGF [69]. In normal brain tissues, TGF-β levels are very low or undetectable. However, the expression of this cytokine is intensified in brain tumors. Depending on the tumor development stage TGF-β can exert either tumor promoting or suppressing effect (Table 1). In advanced stages of glioma, it can stimulate migration and invasion, specifically through neoplastic plexus and ADAM 17 (a disintegrin and metalloprotease) [70]. ADAM regulates proteolysis of extracellular signaling such as cleavage of plasma membrane-anchored forms of ligands. TGF-β is part of the T-helper-3 (Th3) cell response which is characterized by strong immunosuppressive activity, specifically against tumor- infiltrating T-cells. It has been shown to mediate neoplastic plexus in cancer cells through enhanced extracellular matrix production and augmented angiogenesis while simultaneously limiting host cell antitumor immune response. Moreover, TGF-β signaling can cause reduction of NK cells, T-cells and B-cells and macrophage proliferation [71].

In one study, using GBM tumor samples, TGF-β showed the highest mRNA expression levels out of 53 cytokines examined [72]. Though, all three isoforms of TGF-β (TGF-β1, TGF-β2, and TGF-β3) are abundantly expressed in brain cancers, TGF-β2 is the primary isoform highly secreted in GBM and stimulate proliferation of cancer cells. TGF-β2 acts as immunosuppressive cytokine and negatively interferes with the DC maturation and downstream function by lowering MHC class II expression on CD4+ T-cells [73]. Consequently, the IL-12 production by DC slows down, which is necessary to induce subsequent T-cell proliferation and interferon (IFN) production. This cascade of events effectively results in GBM evading the host immune system.

### 4.7. Interleukin-4 (IL-4)

Among the anti-inflammatory cytokines, IL-4 is the most studied in brain cancers. It is mainly produced by T-cells, mast-cells, and basophils [74]. IL-4 plays a significant role in regulating maturation and proliferation of B-cells, mast-cells, and T-cells (Table 1). Like other anti-inflammatory cytokines (IL-5, IL-6, IL-9, IL-10, and IL-13), IL-4 can induce a Th2 response. The Th2 cells are prominent fighters against larger pathogens such as parasites and allergens through humoral immune response and tumor-specific immunity. IL-4 works in conjunction with IL-13 and functions against allergic inflammations through direct activation of Janus kinase (JAK) and STAT3 pathways. There is also evidence that IL-4 inhibits microglial production of pro-inflammatory cytokines, reactive oxygen intermediates and nitric oxide [75].

Due to the overexpression of IL-4 receptor in glioma cell lines and primary specimens compared to the normal brain tissues, it was proposed that IL-4 can serve as a cytotoxic treatment in brain tumor therapy. One pre-clinical study showed around 90% in vitro cell death of U251 and T98G cells upon IL-4 treatment [76]. However, noncancerous cells (such as B-cells, T-cells, and monocytes) showed a minimal response towards this therapeutic approach. Current in vivo, phase I/II studies of IL-4 against recurrent GBM have shown similar results along with limited systemic toxicity [77].

### 4.8. Interleukin-10 (IL-10)

IL-10 is a potent anti-inflammatory and immunosuppressive cytokine released in brain tumor microenvironment [40,78]. IL-10 limits excessive tissue damage caused by the inflammatory response to bacterial and viral infections and exerts its suppressive function by downregulating the expression of a wide range of pro-inflammatory cytokines through activation of the JAK-STAT signaling pathways [79]. High levels of IL-10 expression have been reported in different brain cancers. TAMs and MDSCs discharge large extent of IL-10 that enhances regulatory T-cell (Treg) activation and expansion [80]. Evidence suggests that IL-10 facilitates the shift of T cell’s cytokine production pattern from a T helper 1 (Th1) immune-phenotype (with anti-tumor activity) to Th2 immuno-phenotype (with pro-tumor activity) [81]. Moreover, the expression of IL-10 mRNA has been shown to correlate with the tumor grade. In one study, 87.5% of grades III and IV astrocytomas expressed IL-10 mRNA compared to 4% of grade II astrocytomas [82]. A separate study found that highly invasive glioma tissues expressed IL-10 at higher levels than more localized gliomas [83]. Currently, there is minimal research on IL-10 function in clinical trials.

## 5. Role of Inflammatory Cyclooxygenases in Brain Cancer Development

**Figure 2:**
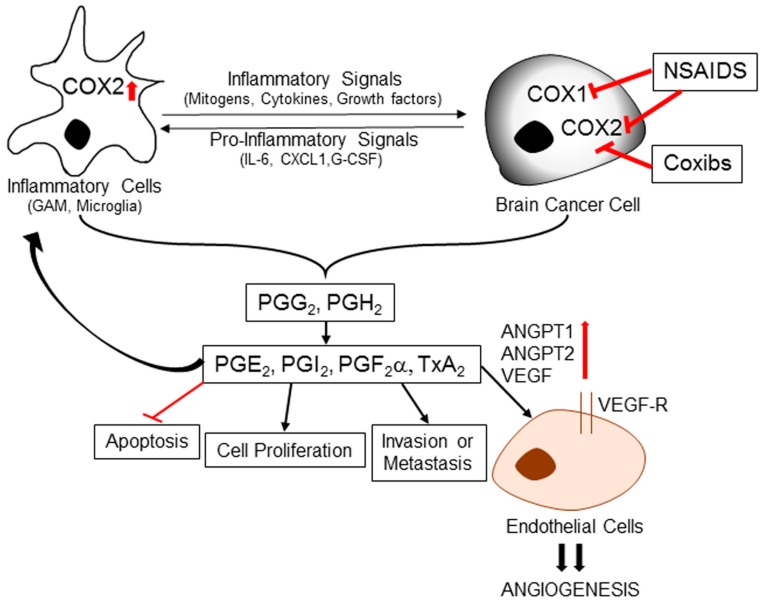
Role of inflammation-induced cyclooxygenase in brain cancer development. Inflammatory signals like mitogens, cytokines and growth factors can promote overexpression of COX-2 in tumor-associated cells immune cells and endothelial cells. COX enzymes catalyze the conversion of arachidonic acid into prostaglandin H2 (PGH_2_) and prostaglandin G2 (PGG_2_), precursors of all prostaglandins (PGs) and thromboxanes (TXs). PGH_2_ is further converted into various effector molecules by specific synthases. These effector molecules can mediate a diverse array of functions, including inflammation, inhibition of apoptosis, cell proliferation, invasion, and metastasis. Some downstream mediators also induce expression of angiopoetins (ANGPT1 and ANGPT2) and vascular endothelial growth factor (VEGF) to promote angiogenic potential of endothelial cells. NSAIDs and COXIBs can inhibit prostaglandin synthesis and thereby prevent pro-carcinogenic downstream effects. NSAIDs: Non-steroidal anti-inflammatory drugs; COXIBs: selective COX-2 inhibitors; CXCL1: CXCL1 chemokine; G-CSF: Granulocyte colony stimulating factor.

Cyclooxygenases play a key role in the inflammatory process, mainly through conversion of arachidonic acid into critical mediators of inflammation such as prostaglandins, prostacyclin, and thromboxane (Figure 2). This synthesis occurs through intermediate molecules prostaglandin G2 (PGG_2_) and prostaglandin H2 (PGH_2_). There are three COX isoforms namely, COX-1, COX-2, and COX-3 [84]. COX-3 is a splice variant of COX-1, which retains intron one and has a frameshift mutation. Several studies confirm that all these COX enzymes share analogous molecular structure and perform similar catalytic reactions. The major distinction between COX-1 and COX-2 is in their tissue expression pattern. A constitutive expression of COX-1 is observed in most normal tissues where it is required for prostaglandin synthesis and maintenance of physiological tissue homeostasis. In contrast, COX-2 is mostly inducible and expressed in a response to various stimuli like inflammatory signals, mitogens, cytokines, and growth factors [85].

COX-2 is constitutively expressed under normal physiological conditions in neuronal cells, and its overexpression has been mainly observed in various inflammatory brain conditions, including multiple sclerosis, amyotrophic lateral sclerosis, Parkinson disease, Alzheimer disease [86]. In these neurodegenerative diseases, COX-2 plays an instrumental role through induction of chronic inflammatory conditions by prostaglandin production (Figure 2). Due to its association with pro-inflammatory activities, COX-2 is a potential contributor in carcinogenesis. Increased expression of COX (mainly COX-2) is identified in the pre-malignant lesions, early stages and late stages of various cancers [87]. Some studies found direct involvement of COX-2 in angiogenesis, tumor invasion, and promotion of tumor cell resistance to apoptosis [88,89].

Several brain cancer studies found COX-2 overexpression and its strong correlation with the tumor grade. In comparison to healthy brain or low-grade glioma, the COX-2 levels are much higher in high-grade gliomas as confirmed by immunohistochemical staining. Moreover, elevated COX-2 levels correlate with earlier recurrence and shorter survival in glioma patients, [90]. According to Shono et al. high COX-2 levels are markers of poor prognosis in brain cancer patients especially those with GBM. Though, how exactly COX-2 contributes to brain cancer pathogenesis is still unclear, many studies have confirmed a direct correlation between COX-2 level and metastatic and invasive potential of brain cancer cells. COX-2 can promote EMT by stimulating the release of proangiogenic prostaglandins. These observations implicate that along with the promotion of inflammatory conditions in tumor microenvironment COX-2 is involved in multiple other tumorigenic mechanisms in brain cancer development (Figure 2).

Considering the invaluable role of COX-2 in brain cancer progression, several groups have attempted to examine the effect of COX-2 specific inhibitors against brain cancers. One of those studies performed on glioma cell lines with a specific COX-2 inhibitor (NS-398), resulted in enhanced apoptosis and attenuated proliferative and invasive potential [91]. In another study, Kang et al. demonstrated that the selective COX-2 inhibitor celecoxib sensitized the U87MG glioblastoma cells to radiation resulting in reduced proliferation and angiogenesis accompanied a downregulation of angiopoietin-1, angiopoietin-2, and VEGF [77]. The median survival of mice with intracranial implantation of glioma cells was also significantly higher (41 days) in the combination group (treated with Celecoxib and radiation) with extensive tumor reduction than the group with irradiation alone (18 days). Several other studies, conducted with different selective COX-2 inhibitors (e.g., Meloxicam) on a panel of glioma cell lines (e.g., D384, U87, and U251), reconfirmed the rationale of targeting COX-2 to induce radiosensitization in brain cancer cells [92]. The anti-angiogenic potential of COX-2 inhibitors was also confirmed in studies above.

## 6. Inflammation-Induced Dysregulated Immune Response: Role of Signal Transducer and Activator of Transcription 3 (STAT3)

**Figure 3:**
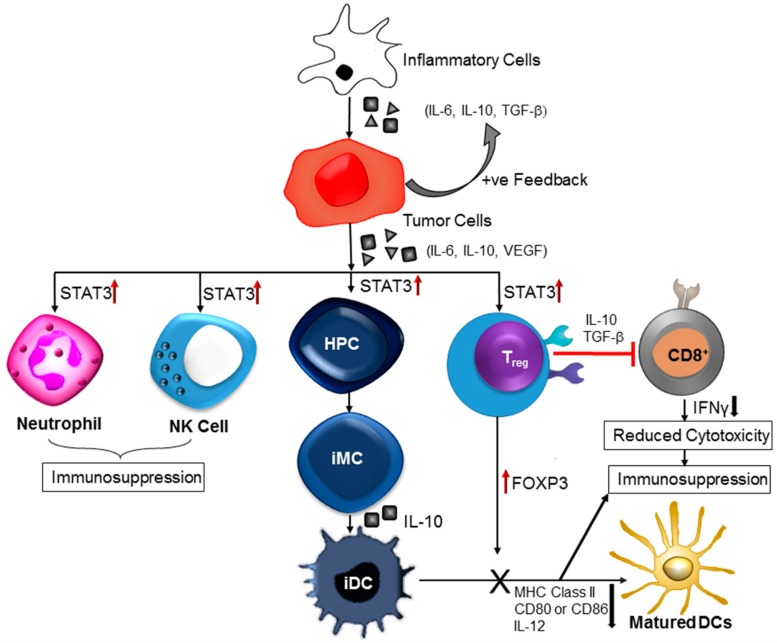
Inflammation-induced immune dysregulation contributes to brain cancer pathogenesis. Cytokines released by inflammatory cells cause upregulation of signal transducer and activation of transcription 3 (STAT3). Increased STAT3 activity negatively affects the immune responses in the tumor stroma. Further, STAT3 signaling in haematopoietic progenitor cells (HPCs) cause generation of immature myeloid cells (iMC) followed by formation of immature dendritic cells (iDC). Dendritic cell (DC) maturation is prevented due to STAT3-mediated reduction in the expression of MHC class II molecules, CD80, CD86, and IL-12. Persistent activation of STAT3 in natural killer (NK) cells and neutrophils inhibits the tumor killing activity of those effector cells. Tumor-associated regulatory T (Treg) cells exhibit increased level of forkhead box P3 (FOXP3), transforming growth factor-β (TGFβ) and interleukin-10 (IL-10), these factors restrain CD8+ effector T-cells and DC maturation.

Immune regulation has profound effects on glioma and other brain cancers which result in a paucity of immunological danger signals necessary for immune activation, the increased concentration of immunosuppressive factors and stacking of immunosuppressive cells in the tumor microenvironment. Escaping from natural immune surveillance is a critical characteristic attributed to most brain cancers. Though, innate immune cells (macrophage, natural killer cell, neutrophil) heavily infiltrate into tumor sites but remain dysfunctional and unable to attack the surrounding cancer cells. Cancer immunologists have been working on this dilemma for a long time. However, some cellular and molecular mechanisms have been identified that mediate this extrinsic tumor suppressing function (Figure 3). STAT3 is one of the central players in this tumor-induced immune deregulation [93,94]. STAT proteins function as cytoplasmic transcription factors that primarily mediate signaling from tyrosine kinases, including growth factors and cytoplasmic enzymes. During activation, STAT proteins are phosphorylated on carboxy-terminal tyrosine residues and undergo dimerization through reciprocal phosphotyrosine-Src Homology 2 (SH2) interactions. The resulting homo- and heterotrimers translocate from cytoplasm to nucleus and bind DNA along with other cofactors. Sequence specificity plays a significant role in the interaction between STAT and STAT-binding nuclear proteins. Any structural modifications (change in amino acid sequence) of these proteins diminished the functional activity of STAT and its transcriptional regulation. STAT-mediated transcriptional regulation is highly dependent on its upstream signaling mechanisms. For instance, IFN activate the STAT-1-mediated transformation whereas IL-6 induces the STAT3 activity [95,96]. Several studies have found a persistent STAT3 activity in various brain cancers [97,98,99]. Though the detection rates obtained from retrospective, immunohistochemistry-based studies varied (9%–83%), STAT3 over-activation is considered to be a precipitating factor in most brain cancers (Figure 3). Some groups have reported a correlation between brain tumor grade and STAT3 activation levels. Rahaman et al. have indicated that 90% of human GBM tumors and GBM cell lines are positive for constitutive STAT3 activation [100]. STAT3 over-activation was also observed in anaplastic astrocytomas and other brain cancers.

The constitutive activation of STAT3 in various brain cancers, including glioblastoma may result from aberrant upstream signaling or defective negative regulation. Convergence of STAT3’s position with different oncogenic signaling pathways is one of the main reasons for its prevalence in glioblastoma. Platelet-PDGFR, heregulin-2/neuregulin receptor (Her2/Neu), EGFR, IL-6R/glycoprotein 130 (gp130), c-Met, Abelson leukemia protein (ABL), and Src tyrosine kinase are the main oncogenes involved in STAT3 activation [95]. Therefore, the overexpression of upstream growth factor receptors/signaling kinases and functional mutations result in persistent STAT3 activation. Sometimes, STAT3 is also activated due to a disruption of normal counter-regulatory mechanisms. An Inflammatory microenvironment almost always accompanies with overproduction of cytokines that may subsequently induce abnormal autocrine or paracrine signaling of cell surface receptors involved in STAT3 activation. Several glioblastoma studies have reported elevated levels of IL-6, IL-10 and TGF-β along with higher STAT3 activity [96]. IL-6 and IL-10 family cytokines are essential STAT3 regulators. The receptor of IL-6 family cytokines shares a common feature-gp130 receptor-β subunit. Binding of IL-6 to its respective receptor mediates gp-130 homodimerization/heterodimerization that ultimately triggers the activation of associated kinases JAK1, JAK2 and subsequent tyrosine phosphorylation of gp130 [95]. For STAT3 activation, the phosphorylation of tyrosine residues in the cytoplasmic tail of gp130 is crucial [101].

Increased STAT3 activity is a negative regulator of immunological danger signals (Figure 3). Different experiments have shown that inhibition of STAT3 activity can upregulate many pro-inflammatory cytokines and chemokines [102]. Immune cell infiltration and activation were observed in tumors expressing a dominant-negative STAT3 mutant protein. There is a negative correlation between STAT3 activity levels (natural or induced) and immune cell migration and infiltration into tumors (Figure 3). An oncogenic fusion protein, paxillin-3-forkhead (PAX3-FKHR) interacts with STAT3 to alter the transcription of various immune-stimulating cytokines and chemokines, and MHC class II molecules in tumor cells. STAT3 mediated downregulation of pro-inflammatory cytokines, and chemokines production is one of the contributing factors to the suppression of immunological response in brain tumors (Figure 3). Another important mechanism of STAT3 mediated immunosuppression is the inhibition of DC maturation (Figure 3). DCs that are present in tumor microenvironment are mainly immature and characterized with an inadequate level of MHC class II complexes and insufficient co-stimulatory signals such as CD80 and CD86, and IL-12 [103]. These DCs can also induce immune tolerance along with their incapability in activating antigen-specific CD8+ T-cells. Some other studies have also confirmed the existence of abnormal STAT3 activity that negatively affects functional maturation of DCs through suppression of MHC class II expression and co-stimulatory molecules like IL-12. Other co-players in the process of STAT3-associated inhibition of DC maturation are VEGF and IL-10. Tumor-induced or IL-10-induced inhibition of DC functional maturation can be abrogated by blocking of STAT3 signaling [100].

Interestingly, two studies have reported the mitochondrial translocation of post-translationally modified STAT3 protein in lung cancer cells [104,105]. Specifically, the serine 727 phosphorylated STAT3 (Ser727-P) and CREB-binding protein (CBP)-acetylated STAT3 were shown to interact with the pyruvate dehydrogenase complex E1 (PDC-E1) and facilitate an efficient electron transport chain (ETC) activity, accelerated conversion of pyruvate to acetyl-CoA, elevated mitochondrial membrane potential. Moreover, STAT3 in mitochondria stabilized the permeability transition pore (MPTP) through interaction with cyclophilin D and blocked the MPTP-mediated cytochrome-c release, thereby preventing initiation of apoptosis [106]. Many brain cancers show characteristic metabolic shifts towards aerobic glycolysis and extensive utilization of TCA cycle intermediates, and the constitutionally active STAT3 may play such modulatory roles in glioma metabolism.

## 7. Role of NF-κB in Inflammatory Mechanism of Brain Cancer Development

**Figure 4:**
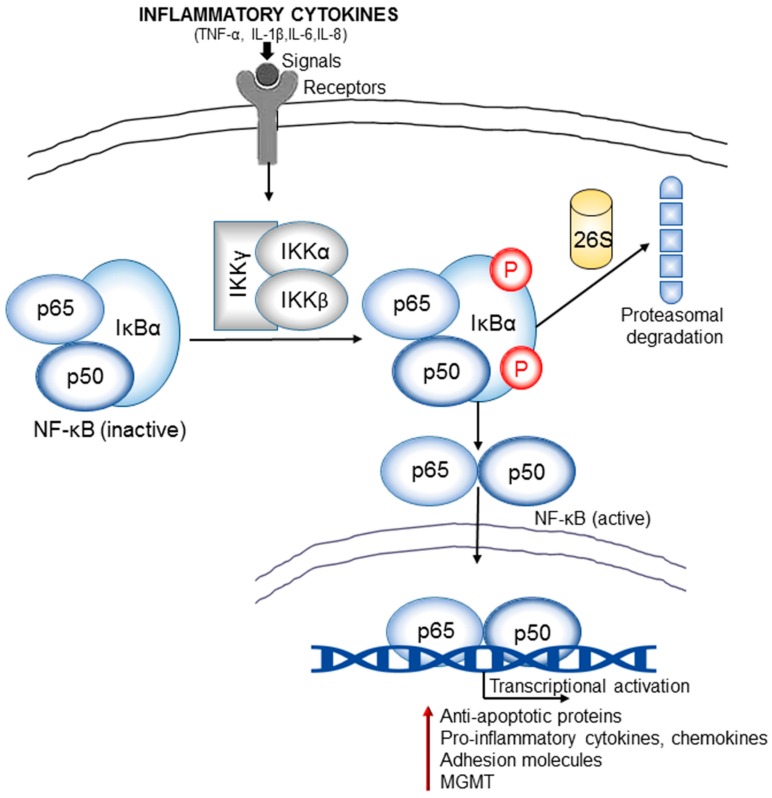
Inflammation and NF-κB signaling cross-talk is an important link in brain cancer pathogenesis. The inflammatory cytokines (TNF-α, IL-1β, IL-6, and IL-8) released in the tumor microenvironment generate signals that lead to activation of IκB kinases (IKKs). IKKs induce phosphorylation and subsequent proteosomal degradation of NF-κB inhibitor, IκBα. The resulting free NF-κB (e.g., heterodimer of p65 and p50 subunits) then translocates to the nucleus and activate the transcription of various target genes that encode anti-apoptotic proteins (e.g., B-cell lymphoma 2/ Bcl-2), pro-inflammatory cytokines and chemokines, adhesion molecules (integrins and cadherins), proteases (MMPs), DNA repair proteins such as the MGMT (*O*^6^-methylguanine DNA methyltransferase). Many of these regulators contribute significantly to the oncogenesis of gliomas.

NF-κB is a family of transcription factors that regulate the expression of various inflammatory, apoptotic, and oncogenic mediators [107]. Structurally, NF-κB is made of homodimers and heterodimers of the five members of the Rel (reticuloendotheliosis oncogene) family, including NF-κB1 (p50/p105), NF-κB2 (p52/100), RelA (p65), and c-Rel. It is present in an inactive form within the cytoplasm of unstimulated cells through its interaction with the inhibitor IκBα (inhibitor of kappa B). Various stimuli like cytokines or DNA damage may initiate the activation process of this transcription factor which begins with phosphorylation of IκBα by activated IκB kinases (IKKα or IKKβ) followed by a K48 ubiquitin-mediated proteasomal mechanism. The resulting free NF-κB now translocates to the nucleus and transcriptionally activate various downstream target genes (Figure 4). NF-κB is either upregulated or constitutively expressed in many cancers including the brain cancers [108]. Several immunohistochemical staining experiments have confirmed increased expression of NF-κB in glioma cells in comparison with healthy brain cells [109,110]. A correlation was also found between NF-κB expression levels and histological grades of gliomas. Higher-grade glioma showed more cytoplasmic and nuclear staining with phospho-p65 antibody than the lower grades. Other studies reported a constitutive NF-κB activation and increased nuclear localization of the p65 and p50 subunits in GBM in comparison to normal astrocytes [111].

Multiple stimulatory pathways can mediate the NF-κB activation. In brain cancer models, mostly two distinct mechanisms initiate NF-κB signaling: one is EGFR signaling and another, the deletion of the NFKBIA gene that encodes IκBα. Brain cancers also harbor higher levels of EGFR gene amplification and mutations that eventually lead to increased level of both wild-type (EGFRwt) and mutant EGFR. The most common mutant form of EGFR in brain cancer is EGFRvIII which exhibits more oncogenic potential than EGFRwt. Both EGFRwt and EGFRvIII can activate NF-κB but through distinct mechanisms [110]. EGFRwt activates the NF-κB in glioma cells via an SHP-2 (SH2-containing tyrosine-specific protein phosphatase) and Gab1 (Growth factor receptor-bound protein 2/GRB2 associated binding protein1)-dependent pathway, and via a PLCγ and PKCε-dependent pathway. Whereas mutant EGFR (EGFRvIII)-mediated NF-κB activation involves receptor-interacting protein (RIP1, RIPK1) and mTOR. Along with these, the TRADD, a major adaptor in TNFα-mediated activation of NF-κB, is commonly expressed at high levels in GBM and confers worse prognosis [112]. NF-κB activation is originally a part of body’s immune defense against cancer development. Upon activation, NF-κB induces the accumulation of cytotoxic immune cells at the tumor site followed by the killing of malignant cells. However, its constitutive activation is frequently observed in many cancers, including gliomas which can be followed by a diverse array of pro-malignant functions. It has been found that during malignancy, constitutively activated NF-κB remains unable to activate proper immunosurveillance mechanisms against aberrant cells. These escaped cancer cells outperform the immune system and contribute to the formation of a chronic inflammatory condition. The proof of concept that an inflammatory tumor microenvironment is frequently associated with constitutive NF-κB activity and exerts pro-tumorigenic effect is validated by some observations where patients with chronic inflammatory diseases are more prone to develop cancer.

The primary role of NF-κB in brain tumorigenesis process appears to be directly related to its involvement in the regulation of apoptosis signaling. Once translocated into the nucleus, it can transcriptionally activate various anti-apoptotic genes including Bcl-2 (B-cell lymphoma 2) and survivin [113]. Several studies have confirmed significantly higher levels of antiapoptotic proteins such as Bcl-2, TRAF1 (TNF receptor-associated factor 1), and survivin in human astrocytic tumors compared with normal brain tissues, which are mostly attributed to an abnormal transcriptional activity of NF-κB through a TNF/TNF-receptor/TRAF2 activation [114]. Interestingly, TNF has a double standard role in cell physiology. Depending on the circumstances, it can either initiate apoptotic cell death or can promote cell survival. There are multiple intermediate components involved in the TNF receptor-mediated signaling cascades: TRAF2 (TNF receptor-associated factor 2), FADD (FAS associating protein with death domain), TRADD (TNF receptor-associated protein with death domain). Among these, the TRADD and FADD cause activation of apoptotic mechanisms and TRAF2 induce nuclear translocation of NF-κB followed by transcriptional activation of antiapoptotic proteins.

The aberrant NF-κB signaling and chronic inflammatory conditions in glioma are intricately linked to each other. There is a vicious cycle that incorporates inflammation, NF-κB signaling, and tumorigenesis. For instance, an inflammatory condition is almost always associated with an extensive release of different pro-inflammatory cytokines such as TNF-α which in order can promote the activation of NF-κB. Activated NF-κB then instigates the release of more inflammatory mediators through transcriptional activation. Several studies have confirmed the role of NF-κB in the induction of inflammatory conditions through facilitating the release different inflammatory cytokines such as IL-6, and chemokines, IL-8, adhesion molecules, MMPs, COX-2, and iNOS (inducible nitric oxide synthase) [115]. These cytokines are involved in tumorigenic signaling pathways that drive pre-cancerous cells towards malignant transformation with unbridled growth potential and increased invasiveness. Some of these cytokines can also activate other transcription factors such as the STAT3 which in turn cooperate with NF-κB to stimulate a pro-survival signaling. In brain cancer studies, a strong correlation was observed among NF-κB activation, IL-6 overexpression, and poor patient survival. It has been found that the inflammatory condition in glioma can stimulate recruitment of macrophage/microglia cells in the tumor microenvironment. These macrophages then produce IL-6 by the aid of over-activated NF-κB and initiate pro-survival signaling, which also incorporates STAT3. The production of IL-8, a chemokine highly overexpressed in glioma, is also regulated by NF-κB. IL-8, a member of CXC chemokines, is secreted during inflammation by activated monocytes and macrophages. It stimulates the migration of pro-inflammatory factors such as T lymphocytes, neutrophils, and basophils towards tumor microenvironment.

NF-κB is also involved in the transcription regulation of *O*^6^-methylguanine DNA methyl transferase (MGMT) [116]. MGMT is a DNA repair enzyme that removes alkyl groups from DNA bases, particularly at the *O*^6^-position of guanine, which is the most nucleophilic site of DNA to undergo alkylation by various endogenous and exogenous metabolites. Most of the alkylating agents such as the chloroethylnitrosoureas (CENUs) or temozolomide (TMZ) exert their cytotoxic effects through alkylation of the *O*^6^-guanine which results in intratumoral mutations or DNA cross-linking and cell cycle arrest. Overexpression of MGMT is a well-established resistance mechanism against these alkylating drugs in different brain cancers [117]. Moreover, glioblastoma patients with high MGMT expression show poorer prognosis than those with low or negative expression [118]. MGMT transcription is regulated by a group of transcription factors and the methylation status of its promoter. Some recent findings have shown that constitutive activation or overexpression of NF-κB is responsible for augmented production of MGMT [116]. A few of NF-κB binding sites have been identified in the MGMT promoter regions; one is at −766 (MGMT-κB-1) and the other one is at −90 (MGMT-Κb-2). A previous study showed that transient transfection of human embryo kidney HEK293 cells with the NF-κB subunit p65 could induce a 55-fold increase in MGMT expression, whereas the addition of the NF-κB inhibitor δNIκB (N-terminal domain of IκBα) abrogated the augmented expression of MGMT [116].

## 8. Inflammation, Oxidative Stress and Genetic Instability in Brain Cancer Progression

**Figure 5:**
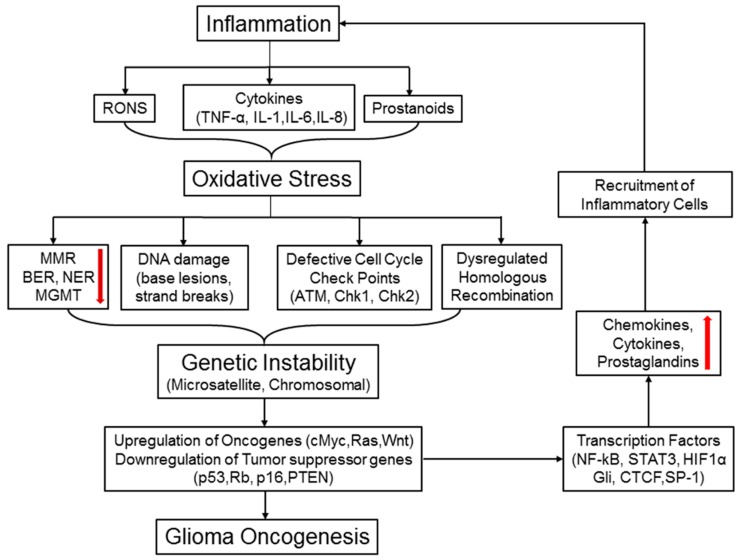
Inflammation, oxidative stress, and chromosomal instability: a vicious cycle that promotes brain tumorigenesis. Inflammatory cells produce various reactive oxygen and nitrogen species (RONS) and other factors, such as cytokines, chemokines, and prostanoids. All these mediators promote oxidative stress in cells leading to deregulated DNA repair systems MMR (DNA mismatch repair), NER (nucleotide excision repair), BER (base excision repair), and MGMT), increased DNA damage, defective cell cycle checkpoints, and dysregulated homologous recombination (HR) pathway. Oxidative stress-induced MMR silencing often causes formation of error-prone microsatellites in the coding regions of various genes and repeated DNA sequences, termed as microsatellite instability (MSI). RONS-mediated DNA damage and strand breaks along with abnormal mitotic checkpoints and homologous recombination (HR) collectively engender genetic instability (GI). Both MSI and GI can spawn random genetic diversification in precancerous cells. Clones harboring the optimal combination of activated oncogenes and inactivated tumor suppressor genes will eventually proceed to initiate malignancy. Upon activation, some oncogenes may turn on the transcription factors like NF-κB, STAT3, and HIF-1α, followed by production of pro-inflammatory cytokines and chemokines.

Inflammation is inherently linked to oxidative stress induction culminating in increased release and accumulation of reactive oxygen and nitrogen species (RONS) at the damaged sites. The RONS, highly reactive radicals with unpaired valence shell electrons, are important players in the innate immune system. Upon stimulation by cytokines, macrophages release superoxide (O_2_.) and hydrogen peroxide (H_2_O_2_) through an NADPH (nicotinamide adenine dinucleotide phosphate)-dependent oxidative burst. Macrophages also generate nitric oxide (NO), an important signaling molecule in modulating vascular tone, which can combine with O_2_ to form peroxynitrite (ONOO-). Neutrophils and eosinophils can combine H_2_O_2_ with halogens to generate hypochlorous acid (HOCl) and hypobromous acid (HOBr). These different reactive oxygen, nitrogen, and halogen species can react with lipids, proteins, and nucleic acids to form a complex array of reaction products that may result in genomic instability (Figure 5). Here, we discuss some inflammation and oxidative stress-mediated genetic and epigenetic mutations involved in brain cancer progression (Figure 5).

### 8.1. Inflammation and Oxidative Stress Induced Genetic Mutations in Brain Cancers

Multiple genes are frequently mutated in different brain cancers, and these mutations play a significant role in tumor initiation and progression. p53 is one of the highly mutation-prone genes found in brain tumors. Mutations can be of different types, and in p53 the most common mutation is single-base changes (85%) [119]. Among them, transition mutations (GC to AT or AT to GC) comprise ~65% of the substitutions. A significant number (~30%) of the GC to AT transitions are within a CpG dinucleotide, and CpG dinucleotides in the p53 gene are often methylated [120]. Therefore, a significant number of p53 mutations arise from the hydrolytic deamination of 5mC to thymine, a base change that is hard to repair. The spectrum of single-base mutations found in the p53 gene outside of CpG dinucleotides in brain tumors is consistent with cycles of endogenous damage and repair cycles.

Oxidative stress often causes DNA damage, which is followed by mutagenesis. 8-oxo-2′-deoxyguanosine (8-oxodG) is one of the prevalent mutagenic DNA oxidation damage products found in glioblastoma [121]. While oxidative DNA damage occurs in all metabolically active cells, levels of 8-oxodG in glioblastoma tissues are reported to be roughly twice those found in normal brain tissues. The increase in oxidation is accompanied by a reduction in total antioxidant capacity. Increased 8-oxodG in GBM tissues leads to enhanced phosphorylation of histone H2AX (γH2AX) and induction of a DNA damage response that signals to p53. While in the tumor microenvironment, ROS from outside of the cell contributes to augmented DNA damage and molecular alterations within the cell can also increase oxidative stress. Glioblastoma cells with specific EGFRvIII mutations have increased 8-oxodG and upregulate DNA repair genes in response to increased DNA damage [122]. Presumably, increased signaling through EGFR results in increased oxidative stress, DNA damage, and further promotes genetic instability.

Single-base oxidative damage like 8-oxodG is primarily repaired by the base excision repair (BER) pathway. Although glioblastoma cells experience enhanced oxidative damage within the tumor microenvironment, the levels of multiple glycosylases of the BER pathway are significantly downregulated in astrocytoma Grades II to IV. Expression of the 8-oxoguanine DNA glycosylase 1 (OGG1) involved in 8-oxodG repair is reduced by more than an order of magnitude. Interestingly, patients with high EGFR expression and small relative BER capacity had longer survival times, suggesting that the relationship between DNA damage and repair in glioma might extend beyond tumor initiation and could uncover targets for selective chemotherapy.

### 8.2. Inflammation and Microsatellite Instability (MSI)

Microsatellite instability is characterized by increased genetic instability due to mutations or epigenetic silencing of mismatch repair proteins [123]. The mismatch DNA repair (MMR) proteins function to eliminate mis-incorporated bases and remove larger insertions/deletions and maintain replication fidelity. When MMR is non-functional, the DNA replication becomes faulty, and errors accumulate throughout the genome. These errors preferentially affect genes (such as TGFβRII, IGF-2R, and BAX) that contain microsatellites in the coding regions. Microsatellites are short repetitive nucleotide sequences present in DNA, intrinsically unstable and therefore, prone to be copied incorrectly during DNA replication. Inflammation-mediated downregulation of MMR proteins can occur through several mechanisms. One such mechanism involves HIF-1α; inflammatory cytokines (TNF and IL-1β) often induce HIF-1α in tumor microenvironment which along with PGE2 and RONS downregulate the MMR proteins MSH2 and MSH6 by displacing c-Myc (cellular myelocytomatosis oncogene) from MSH2/MSH6 promoters [124].

Studies have confirmed a direct role of oxidative stress on MMR inactivation that often leads to carcinogenesis. For instance, H_2_O_2_ can oxidize the MMR members, NO can upregulate of cytosine-DNA methyltransferase to trigger promoter silencing and loss of gene expression of hMLH1 (human MutL homolog 1). Several clinical studies have reported that a significant number of malignant brain tumors show negative immunohistochemical staining for hMLH1 [125]. High-grade gliomas are often associated with hMLH1 promoter hypermethylation. Moreover, various inflammatory brain diseases such as Alzheimer’s exhibit methylation of hMLH1 gene promoter [126]. These findings indicate that MSI can be detected early in premalignant tissues (without dysplasia) of brain cancer patients, suggesting that inflammation-mediated inactivation of the MMR system can be a diagnostic marker in brain cancers.

Other than the MMR pathway, the BER system can also contribute to the generation of MSI. Studies found a positive correlation between overproduction of BER enzymes and MSI in glioblastoma [127]. This process can be explained from observations where ROS production can induce overexpression of BER enzymes, and the coding regions of these proteins contain the consensus sequence for NF-κB binding. IL-6 is another factor that can generate MSI mainly through altering the nucleotide excision repair (NER) pathway. NER system performs the repair of DNA lesions caused by UV radiation, mutagenic chemicals, and chemotherapeutic agents. Evidently, IL-6 induces hypermethylation of hHR23B, which is a key nucleotide excision repair component. HIF-1a induces the microRNA-373 that downregulates the expression of the NER component RAD23B (UV excision repair protein RAD23 homolog B).

### 8.3. Inflammation and Chromosomal Instability

Chromosomal instability (CIN), often characterized by abnormal segregation of chromosomes, is one of the key events that can lead to tumorigenesis and cancer progression. Though the exact molecular mechanism of CIN induction in tumor cells is still unclear, a major feature of this defect is dysregulation in mitotic checkpoint proteins. Mitotic checkpoints are the guardian of the genome and act on ensuring genetic integrity throughout the cell’s life cycle. Any damage or mutation inflicted on DNA would cause activation of the checkpoint proteins followed by initiation of DNA repair. The whole cell cycle progresses under a strict surveillance of these regulatory proteins, and any impairment in this function may lead to chromosomal mis-aggregation and aneuploidy.

p53 is considered to be one of the most important protector of genomic integrity and chromosomal stability. In its wild-type form, p53 mediated pathways work on protecting cells from malignant transformation by inducing cell cycle arrest, DNA repair and/or apoptosis. Loss of p53 or its mutation may result in defective mitotic checkpoints and increased CIN through an aberrant exit from mitotic arrest. Several studies in different cancer models have reported that cells harboring mutant p53 undergo extensive chromosomal mis-segregation and abnormal cytokinesis that almost always lead to aneuploidy and/or tetraploidy formation. Moreover, the aberrant chromosomal division may cause activation of different oncogenes or inactivation of various tumor suppressor genes. Brain tumorigenesis is often associated with chromosomal instability and p53 inactivation [128]. Among the patients with higher grade gliomas, approximately 70% show coexistence of p53 mutation and chromosomal instability phenotype. A significant number of p53 mutations arise from methylation of the CpG dinucleotides in the p53 gene. The inflammatory cytokines (IL-6) and oxidative stress mediators increase the activity of DNA methyltransferase. Moreover, NO and its various derivatives that are produced in inflammatory tumor microenvironment can lead to inactivation of functional p53.

Mitotic arrest deficient 2 (Mad2), an essential spindle checkpoint protein, is another important protein involved in the induction of CIN in glioma [129]. Mad2 is overexpressed in several tumor types, including glioblastoma. Elevated Mad2 can produce a hyperactive spindle checkpoint and thereby altering the sequence of mitotic events and the accuracy of chromosome segregation. Inflammation has a direct role in overexpression of this protein. It is found that NO can induce hyperphosphorylation of RB protein enabling the release of E2F/DP transcription factors, which in turn can instigate transcriptional activation of Mad2 gene expression.

Inflammatory brain tumor microenvironment is heavily infiltrated with macrophage, microglia and other lymphocytes that release an excessive amount of RONS. These RONS often induce DNA double-strand break (DSB) in surrounding cells, which is followed by activation of DSB repair mechanisms: homologous recombination (HR) and non-homologous end joining. Induction of DSB impairs genome integrity since the non-homologous end-joining pathway is an intrinsically error prone and creates small regions of non-template nucleotides around the damage sites. Though HR is an error-free DNA repair mechanism in certain cases, excessive uncontrolled HR may promote chromosomal instability and HR deficiency in small regions of non-template nucleotides around the DNA break. Growth factors and chemokines, produced by inflammatory cells in tumor microenvironment, can induce overexpression of c-Myc in different brain cancer cells [130]. c-Myc plays a major role in brain tumor development and progression, mainly by altering the expression of various genes involved in cell growth, apoptosis, and invasion [131]. Moreover, c-Myc also accelerates the intrinsic mutation rate in glioma cells through several mechanisms. For instance, it can induce double-strand DNA breaks, activate the RAS signaling pathways, and induce ROS generation. c-Myc can also lead to aberrant and incomplete DNA synthesis through utilization of cryptic replication origins.

### 8.4. Inflammation and Oxidative Stress-Induced Epigenetic Changes in Brain Cancer

Epigenetic regulation which includes maintenance of appropriate patterns of histone modifications, DNA methylation, and microRNA expression, plays a significant role in normal cellular development and differentiation. Epigenetic dysregulation is one of the glioma-specific mechanisms of tumor initiation and progression [132]. It is now evident that epigenetic abnormalities may precede classical transforming events like mutations in cancer-relevant genes and genomic instability. Disruption of epigenetic machinery, either by mutation, deletion, or altered expression of any of their components, contributes to epigenetic abnormalities followed by activation of tumorigenic pathways. For instance, aberrant promoter methylation of genes may complement mutation or deletion of the second allele, as postulated by the two-hit model for inactivation of tumor suppressor genes, or even provide both hits by methylation of both alleles. Identification of new “epimutations” is rapidly increasing with the availability of more performing technologies. Similar to genetic alterations, tumor-type specific patterns of epigenetic changes are observed. Promoter hypermethylation of the repair gene that encodes MGMT is by far the most frequent epigenetic alteration in glioma [133]. Epigenetic silencing of MGMT can lead to AT to GC transition mutation, and that has become the first epigenetic biomarker in this disease. Many studies have been conducted on MGMT methylation status in various brain tumors, and a direct correlation was observed between methylation of MGMT promoter and tumor type and/or malignancy grade. For instance, around 40% GBM and 80% anaplastic oligoastrocytomas were diagnosed with MGMT promoter methylation while pilocytic astrocytoma and most non-glial brain tumors were mostly negative [13].

Besides the silencing of DNA repair enzyme MGMT, DNA methylation is also responsible for inactivation of several other critical regulators of mitogenic signaling pathways. In GBM the WNT pathway may be activated through promoter methylation of negative regulators such as the WNT inhibitory factor 1, the family of secreted frizzled-related proteins (sFRPs), dickkopf (DKK), and naked (NKDs) genes [134,135]. Another example is the Ras pathway that in a subset of GBM is deregulated by silencing of the negative regulators Ras association (RalGDS/AF-6) domain family members RASSF1A and RASSF10 [136,137]. RASSF1 is methylated in many tumor types and is thought to contribute to ras signaling. On the basis of DNA methylation status, GBM patients are divided into three subgroups. Among these, the glioma CpG island methylator phenotype (G-CIMP) tumors show highest methylation status along with distinct genetic mutations such as IDH1/2 mutations [138].

*Helicobacter pylori* (HP) infection in the human stomach causes chronic inflammation in the gastric mucosa and activates multiple oncogenic pathways through induction of epimutations [139]. Very few studies have been conducted in brain cancer models to understand the significance of inflammatory conditions on epigenetic dysregulation. However, the inflammatory conditions can induce epigenetic alteration through RONS production [140]. The tumor microenvironment in glioblastoma promotes the infiltration of immune cells, including eosinophils and neutrophils. Upon activation, both initiate an oxidative burst that generates H_2_O_2_, HOBr, and HOCl. These inflammation-mediated ROS can induce DNA damage and may result in the formation of different types of DNA damage products such as 5-chlorocytosine (5ClC) and 5-bromocytosine (5BrC) [141]. Both 5ClC and 5BrC have been shown in in vitro studies to mimic 5mC and to act as fraudulent epigenetic signals [142]. Therefore, the inflammation-mediated formation of either 5ClC or 5BrC could account in part for the aberrant hypermethylation in glioblastoma.

Proteins that are involved in post-translational modifications of histones undergo frequent undergoing through epigenetic alterations and play important role in gliomas. For example, enhancer of zeste human homolog 2 gene (EZH2), a catalytic component of the polycomb repressive complexes, is overexpressed in different brain tumors [143]. EZH2 can control DNA methylation through facilitating the recruitment of DNA methyltransferases. Large scale studies have identified several other epigenetic regulators such as histone deacetylases (HDAC), histone methyltransferases, and histone methylases that are altered in gliomas and may have associations with glioma oncogenesis [144,145]. The inflammatory conditions in gliomas can influence the histone modification in several ways. Inflammatory bowel diseases have shown differential patterns of histone acetylation and transcriptional activation of various oncogenes. Inflammation associated hydrogen peroxide can enhance histone acetylation and histone acetyl transferase (HAT) activity. Histone modification is also involved in the regulation of both pro- and anti-inflammatory cytokines. Acetylation of histone H3 at the promoters of several cytokines and chemokines after inflammation results in the increased recruitment of NF-κB to these regions which results in increased production of those inflammatory mediators.

MicroRNA alteration is another predominant feature in glioma epigenetics. Many microRNAs show a characteristic expression pattern in brain cancer cells and associated macrophages that are involved in regulation of various essential genes. For example, miR-21 is a frequently altered microRNA in GBM, and its elevated expression is found to be mutagenic [146]. The miR-21 targets a number of tumor suppressor genes, including the p53, programmed cell death 4 (PDCD4), tropomyosin 1, PTEN, and TGF-β. Increased miR-21 expression is correlated with aberrant cell proliferation and invasion along with inhibition of apoptosis [147]. It is also suggested as a biomarker for chemo-resistance in different brain cancer. There is a link between the chronic inflammatory condition and increased level of miR-21. Inflammatory stimuli can increase the expression of miR-21, as evident from experiments where inflammatory cytokines (e.g., IL-6) can induce miR-21 expression in a STAT3 dependent manner. Several other microRNAs have been recently identified that may take part in gliomagenesis such as miR-10b, miR196, and miR-155 [148,149,150]. Both low-grade and high-grade gliomas show upregulation of miR-10b that correlates with downregulation of critical cell cycle regulators. Inflammatory tumor microenvironment may influence the expression patterns of these microRNAs which is evident from studies conducted in gastric cancers. It was observed that HP infection can alter the expression of many microRNAs that are involved in cell proliferation, cell cycle regulation, apoptosis, epithelial to mesenchymal transition and immune response.

## 9. Inflammation-Associated Altered Metabolism in Brain Cancer

Altered metabolism is a predominant phenotype in cancer cells and refers to alterations in the synthesis and/or utilization of important metabolites (e.g., glucose, glycogen, fatty acids, amino acids, and glutamine) by tumor cells. Due to aggressive growth characteristic cancer, cells need to produce an extraordinary amount of energy in the form of ATP. Normal cell produces most of its ATP through mitochondria and a small portion through glycolysis. However, cancer cells utilize aerobic glycolysis to generate most of their energy. This phenomenon is called “Warburg Effect”. Brain tumors also exhibit aberrant glycolysis and increased lactate:pyruvate ratios [151]. In addition to altered glucose metabolism, there are numerous other metabolic abnormalities observed in cancers that play an important role in enhancement of tumorigenesis. For instance, metabolism of amino acid tryptophan. Tryptophan, an essential amino acid, acts as a building block for protein synthesis but also functions as a precursor for other biochemical mediators, including serotonin. However, in a cancer microenvironment, tryptophan undergoes metabolism through the kynurenine pathway (KP), ultimately generating quinolinic acid needed for the synthesis of nicotinamide adenine dinucleotide (NAD**^+^**). Cancer cells always crave for extra NAD**^+^** as it is required for various survival mechanisms including ATP synthesis, intracellular calcium homeostasis, and DNA repair. In the inflammatory microenvironment, oxidative damage to DNA triggers a DNA damage response, including activation of Poly (ADP-ribose) polymerase-1 (PARP-1) which consumes NAD**^+^**. PARP-1 protein is usually not present in normal neurons, but human primary glioblastoma tissues showed positive immunohistochemical staining for PARP-1 [152]. Overexpression of PARP-1 and its constitutive activation is found to be associated with self-renewal properties of glioblastoma-initiating cells.

The first metabolite of the KP is kynurenine. Previous studies have reported that kynurenine can modulate T-cell function through the aryl hydrocarbon receptor (AhR) present on T-cells [153]. Kynurenine acts as an endogenous ligand for AhR and activates the formation of Treg while inhibiting natural killer cells [154]. Activation of the AhR can also promote glioma cell proliferation and invasion through upregulation of TGF-β. While the effect of kynurenine on T-cell function is likely part of an immunomodulatory feedback loop needed for the maintenance of homeostasis, its impact on tumor growth, evasion of immune regulation, and patient survival may be profound. Excessive production of this metabolite is often correlated with increased immunosuppression and decreased patient survival. Kynurenine can be converted to kynurenic acid (KYNA) which acts as an antagonist of glutamate-gated ion channel receptors such as the *N*-methyl-d-aspartate (NMDA), α-amino-3-hydroxy-5-methyl-4-isoxazolepropionic acid (AMPA), and kainate receptors. KYNA exhibits neuroprotective effects by inhibiting glutamate excitotoxicity. It also stimulates proliferation of human glioblastoma cells. Quinolinic acid (QUIN), another metabolite of kynurenine pathway, is extensively used by cancer cells to replenish NAD**^+^** which is utilized in various tumor progressing signaling pathways. It is reported that QUIN can also promote proliferation of glioma cells in culture [155]. Another important perspective of QUIN is its effect on glutamate and glutamate receptor. QUIN can instigate astrocytes to release glutamate, the primary excitatory neurotransmitter. At the same time, it also decreases the brain-derived neurotrophic factor (BDNF) that keeps the neurons healthy. Glutamate-mediated neurotoxicity leads to the destruction of normal brain cells. However, glioma cells have a much higher threshold for glutamate damage. Moreover, glutamate binding to glioma-specific receptors can even increase their proliferation potential that results in expansion of the tumor throughout the brain.

Inflammatory microenvironment of glioma can act as a trigger for the activation of KP. It is reported that inflammatory cytokines present in brain tumor microenvironment (IFN-α, IFN-γ, TNF-α, TGF-β, IL-4, and IL-23) can activate this altered metabolism through induction of different enzymes involved in different steps of KP such as Indoleamine 2,3-dioxygenase (IDO) which converts tryptophan to kynurenine [156]. Several studies have confirmed the increases in the abundance of transcripts encoding different KP enzymes such as IDO, kynureninase or 3-hydroxyanthranilic acid oxygenase following treatment with cytokines [157,158]. Considering the prevalent inflammatory conditions in brain tumor microenvironment along with increased production KP metabolites it is understandable that inflammation contributes to brain tumor progression through induction of altered metabolism.

As stated earlier, aberration of TCA cycle due to IDH mutation is another frequently observed metabolic abnormality in brain cancers, especially in lower grade gliomas [8]. In most cases, IDH1/2 mutations result in a gain of function that promote the conversion of αKG (α-ketoglutaric acid) to d-2HG which, in turn, inhibits the αKG-dependent enzyme activities (demethylases and hydroxylases), ultimately leading to an epigenetic dysregulation through a global increase of cellular DNA and histone hypermethylation [9,11,159]. Moreover, tumors with isocitrate dehydrogenase 1 or 2 (IDH1/2) mutations exhibit increased oxidative stress through changes in NADPH and glutathione content. Persistent levels of redox stress may further contribute to a chronic inflammation and oncogenic transformation in human gliomas. More information on IDH mutations is provided in Section 11.

## 10. Anti-Inflammatory and Immunomodulatory Drugs as Potential Therapeutic Options for Brain Cancer Treatment

The role of the inflammatory microenvironment in cancer progression is well-established. Chronic inflammation leads to infiltration of mononuclear immune cells (including macrophages, lymphocytes, and plasma cells), tissue destruction, fibrosis, and increased angiogenesis. The chronic inflammatory condition can also cause elevated DNA damage, increased DNA synthesis and cellular proliferation, disruption of DNA repair pathways, inhibition of apoptosis, the promotion of angiogenesis and invasion. Due to its indispensable contribution in cancer initiation and progression inflammation has been considered as a potential target in cancer prevention and treatment. A lot of studies have been conducted to treat cancers by using various anti-inflammatory agents. The first of such studies was done by targeting COX-2 with aspirin, a non-steroidal anti-inflammatory drug (NSAID), in colorectal cancer [160]. This study and several other clinical trials have demonstrated that long-term use of aspirin or other NSAIDs can prevent various cancers, including colorectal, esophageal, breast, lung and bladder cancers [161]. Some brain tumor studies have also shown the ability of anti-inflammatory drugs to suppress the growth of cell lines derived from medulloblastoma and glioblastoma multiforme [162,163,164]. One such study was done with flurbiprofen; results from this study showed a significant inhibition of tumor cell growth in a dose-dependent manner and a discernible change in the progression of cells through cell cycle phases. Another recent study was done by Gaist et al. to elucidate the influence of NSAID use on glioma incidence. In that study, authors found that long-term use of low-dose aspirin can reduce the risk of glioma. Initial studies were done with broad-spectrum NSAIDs that can inhibit both COX-1 and COX-2. With the advent of specific COX inhibitors (e.g., Celecoxib), it is now possible to examine the precise role of individual COX enzymes in cancer pathogenesis. Although experiments conducted with both selective and non-specific COX inhibitors showed promising results, but the usefulness of these agents remains controversial due to various toxic side-effects such as gastrointestinal toxicity and cardiotoxicity. Nevertheless, different anti-inflammatory agents are still under investigation to improve the therapeutic benefit to the toxicity ratio. Recent studies are concentrating on evaluating the prospect of combining anti-inflammatory agents with conventional chemotherapeutic drugs. In one such studies, conducted on five different glioma cell lines, COX-2 inhibition was found to enhance the cytotoxicity of chemotherapeutic drugs such as doxorubicin and vincristine [165]. Another study was done by Petersen et al. to evaluate the combined effect of SC-236, a selective COX-2 inhibitor, and radiotherapy in human glioma cell lines with moderate expression of COX-2 [166]. In that study, COX-2 inhibition resulted in a significant reduction of cell survival and cell cycle arrest at G2/M phase.

Though COX-2 is the most frequently evaluated anti-cancer anti-inflammatory target, numerous other targets, such as NF-κB, cytokines/cytokine receptors, chemokines/chemokine receptors, FGF/FGFR (fibroblast growth factor/receptor), and VEGF have also been evaluated. Along with the NSAIDs, several steroidal anti-inflammatory drugs have shown promising results in adjuvant therapy. For instance, corticosteroids have exhibited potent anti-cancer activity when used alone or in combination with chemotherapeutic agents [167]. Another study has demonstrated that pretreatment with dexamethasone can enhance the effects of conventional therapies against animal models of glioma, as well as breast, lung and colon cancers. In fact, co-administration of dexamethasone led to a 2–4-fold increase in the efficacy of carboplatin and/or gemcitabine [168].

Since inflammation-mediated immune dysregulation significantly contribute to the progression of both primary brain cancers and brain metastases, recent studies have focused on evaluating the feasibility of immunomodulatory agents against brain tumors [3]. Abnormal STAT3 signaling in regulatory T-cells upregulate the expression of factors like FOXP3, TGFβ, and IL-10 which in turn, inhibit T cell response [94]. Several monoclonal antibodies have been developed to activate the antitumor T cell response via interference with tumor-associated immunosuppression induced by the immune checkpoint molecules. For instance, nivolumab and pembrolizumab inhibit the T cell co-receptor programmed cell death 1 (PD1) [3]. Once activated, PD1 can initiate a signaling cascade that results in decreased survival and proliferation of CD8+ T-cells, reduced cytokine production, and T cell exhaustion. Anti-PD1 antibodies block the interaction of PD1 receptor with its ligands (PD-L1 and PD-L2) and restore the T cell function that eventually leads to an enhanced antitumor response [169]. Some other immunotherapies (antibodies, toxins, and receptor inhibitors) have been designed to target the overproduced inflammatory and immunosuppressing cytokines and chemokines (e.g., IL-4, IL-10, TGFβ) in brain tumor microenvironment [170]. These therapies showed promising efficacies alone and in combination with chemotherapeutic agents.

## 11. IDH1 Mutations in Lower-Grade Gliomas and Their Consequences to Epigenetic Modifications and Augmentation of Oxidative Stress

Research in the last five years has uncovered the presence of specific mutations in the isocitrate dehydrogenase1 (IDH1, R132H at the active site) in >70% of low to mid-grade gliomas, providing a new impetus for drug discovery [171,172]. IDH1 is a key enzyme of Krebs cycle that converts isocitrate to α-ketoglutarate (α-KG) in an NADPH (a major reducing equivalent) generating reaction [172]. However, the point mutations in IDH1 are characterized by loss of normal function and gain of an unusual new activity of reducing α-KG to d-2HG in an NADPH consuming reaction [173,174], thereby, exacerbating the already high oxidative stress in gliomas. More intriguingly, clinicians and neuro-oncologists have consistently reported superior chemotherapeutic response rates and significantly longer survival of patients with IDH mutations [175,176]. The presence of the oncometabolite d-2HG, at higher steady-state concentrations (up to 25 mM) in human gliomas has a plethora of physiological effects of inducing cell differentiation, lipid peroxidation, cardiomyopathy, neurodegeneration and redox imbalance [177].

As a competitive inhibitor of α-KG dependent reactions, the most important consequence of d-2HG is the induction of DNA hypermethylation, a property that best explains the oncogenesis of grade II and III astrocytomas, oligodendrogliomas and secondary GBM. IDH1 mutations are characterized by both the loss of regular and gain of new enzymatic activity, because of which it reduces α-KG to d-2HG consuming NADPH. IDH mutations in low-grade gliomas have a significantly positive effect on overall survival (hazard ratio, 0.64) with TMZ treatment [178], independent of histologic phenotype, and usually predict the presence of MGMT promoter methylation in 84% of IDH-mutant low-grade tumors [171]. Conversely, the absence of IDH mutations in low-grade gliomas has been found to be predictive of a briefer latency to malignant transformation and a shorter overall survival [178]. The better therapeutic responses observed in patients with IDH1 mutations appear to be closely linked to the epigenetic modifications. This is because, the d-2HG inhibits the α-KG-dependent dioxygenases like TET (ten-eleven translocation), JmjC domain containing KDMs (histone lysine demethylases) that are involved in epigenetic modifications and inactivation of ALKBH (alkylation repair homologs) DNA repair proteins that ultimately lead to hypermethylation of the CpG islands in the global genome [179,180,181]. The expression of CpG Island Methylator Phenotype (CIMP) by d-2HG accounts for major gene expression changes [181] including the silencing of MGMT (*O*^6^-methylguanine DNA methyltransferase), which is a single most important determinant of glioma resistance to alkylating agents [182]. Most IDH1 mutant brain cancers fail to express MGMT protein, largely due to promoter methylation [182], which may explain better therapeutic responses in patients positive for IDH1 mutations.

## 12. Conclusions

Brain cancers are the least understood among all malignancies because of their highly heterogeneous cell populations and complex pathogenesis. There are some common characteristics found in various brain cancers, including multiple genetic and epigenetic abnormalities affecting mostly the tumor suppressor proteins (p53, PTEN, Rb) and DNA repair machinery (MGMT, MMR, BER, NER). Inflammatory microenvironment is another characteristic feature of all brain cancers, and recent studies have convincingly shown a strong link between inflammation and carcinogenesis. The molecular and cellular pathways that link inflammation to cancer progression are diverse and encompass a broad range of components. The key players of inflammation-mediated malignant progression are transcription factors, cytokines, chemokines and infiltrating leukocytes. All these factors are mainly involved in two distinct mechanisms: intrinsic and extrinsic. Intrinsic pathways involve the activation of different classes of oncogenes that drive stimulation of various pro-inflammatory mechanisms and steer the formation of a chronic inflammatory milieu. In contrast, the extrinsic mechanisms arise from an existing inflammatory condition that can promote tumorigenesis either through inactivation of tumor suppressor genes or activation of proto-oncogenes.

Microglia or TAM are ubiquitous in brain cancer microenvironment that constitutes up to 30–50% of the tumor mass. TAM and brain cancer cells interact with each other in a complicated fashion and exert diverse responses. In typical cases, infectious and traumatic conditions of tumor induce the activation of TAM which then produces various pro-inflammatory mediators, including cytokines, chemokines, ROS, and NO. The main purpose of this inflammatory response is to eliminate the causal organism which in this case are tumor cells. However, prolonged or excessive activation of these mechanisms can backfire and may promote tumor cell growth, cell survival, and invasiveness through immunomodulation and activation of different oncogene transcription. STAT3 is the prime transcription regulators that mediate chronic inflammation induced immunosuppression via downregulation of pro-inflammatory cytokine and chemokine production. Several studies have confirmed the overexpression of STAT3 in various brain cancers and its role in the regulation of inflammatory mediators.

An inflammatory tumor microenvironment triggers a release of various cytokines, chemokines and growth factors that in turn instigate multiple signaling pathways. These signals enable cancer cells to maintain a sustained growth, avoid apoptosis, and achieve metastatic potential. For instance, TGF-β is overexpressed in glioblastoma that causes immunosuppression and stimulation of cellular proliferation. IL-6 is another cytokine frequently elevated in different brain cancers and involved in modulation of immune responses. It also induces angiogenic activation, cell proliferation, and anti-apoptotic signaling. In addition, there are similar other cytokines such as IL-8, IL-10, TNF-α, that are reported to play critical roles in inflammatory tumor pathogenesis. Chemokines are another group of soluble immune and inflammatory mediators often released during cancerous conditions and attract different myeloid derived cells in the tumor microenvironment. These are abundantly present in gliomas and other brain cancers and execute their chemo-attraction function, which initiates recruitment of microglia and macrophages to the tumor site. Thus, cytokines and chemokines are essential components in brain cancer progression, however, their relevance to therapy is yet to be realized.

NF-κB is considered to be an important link between inflammation and tumorigenesis. As a transcription factor, it is regulating the expression of various genes, which are involved in inflammatory response, cell proliferation, and apoptosis. Most brain cancers show abnormal accumulation of NF-κB and its constitutive activation. Brain cancer pathogenesis accompanies a vicious cycle associating inflammatory condition, NF-κB transcription activation/nuclear translocation and oncogenic signaling. Therefore, NF-κB can be a critical target brain cancer treatment. The oxidative stress mediators are also mutagenic and can generate genetic and epigenetic alterations followed by microsatellite and chromosomal instability (Figure 5). Accumulating evidence suggests that RONS can directly or indirectly affect DNA repair machinery and cell cycle checkpoints as well, thereby precipitating genomic diversification and intra- and inter-tumoral heterogeneity in brain cancers (Figure 5). Finally, the IDH1 mutations prevalent in lower grade gliomas and recurrent glioblastomas appear to induce their oncogenic effects, at least partly, by heightening the oxidative stress and inflammatory responses. Whether we can exploit the information gleamed from many of the processes outlined here for improved therapy of brain tumors remains a valid question and merits further investigation.

## Figures and Tables

**Table 1:** Role of inflammatory cytokines in brain cancer pathogenesis.

Cytokines and Chemokines Found in Brain Tumor Microenvironment			
Cytokines	Normal Function in Brain	Functions in Brain Tumor Pathogenesis	References
Pro-inflammatory			
IL-6	Maturation of B-cells into antibody producing cells Involvement in neurogenesis upon tissue injury	Induction of cancer cell proliferation and invasiveness Promotion of angiogenesis and resistance to apoptosis	[41,42,43,44,45,46]
IL-8	Chemo-attraction of neutrophils, basophils and T-cells Induction of MMP production Inhibition of endothelial cell apoptosis	Promotion of angiogenesis, migration/metastasis Association with glioma aggressiveness and invasion	[47,48,49,50]
IL-1β	Induction of other pro-inflammatory cytokine release Involvement in immune response	Promotion of invasion Association with migration/metastasis Involvement in anti-apoptotic signaling	[51,52,53,54,55,56]
MIF	Involvement in inflammatory immune response Regulation of p53 function	High expression in brain tumor initiating cells Influence on tumor cell proliferation and apoptosis	[57,58,59,60,61,62]
TNF-α	Stimulation of T-cell growth Enhancement of monocyte, granulocyte, and NK-cell mediated cytotoxicity Influences dendritic cell maturation	Promotion of glioma cell invasion and angiogenesis Promotion of migration and metastasis Association with cachexia	[63,64,65,66,67,68]
Anti-inflammatory			
TGF-β	Inhibition of B- and T-cell proliferation Prevention of DC maturation Inference with cytotoxic T cell development	Promotion of tumor growth Immunosuppression Stimulation of GBM cell migration and angiogenesis Downregulation of MHC class II expression on CD4+ T-cells	[73,74,75,76,77,78,79]
IL-4	Processing of anti-inflammatory effects Regulation of allergy response through JAK and STAT pathways Maturation and proliferation of B-cells, mast-cells and T-cells	Major player in the immune-evasion mechanisms of glioma microenvironment Inhibition of micro-glial production of pro-inflammatory cytokines	[74,75,76,77]
IL-10	Suppression of immune response Downregulation of type 1 T helper cell (Th1) cytokines	Overexpression in GBM Inhibition of T-cell proliferation Downregulation of MHC class II Enhancement of tumor progression	[40,78,79,80,81,82,83]
